# Supplementary material for: EBF1 Negatively Regulates Brassinosteroid-Induced Apical Hook Development and Cell Elongation through Promoting BZR1 Degradation
Source: Int J Mol Sci. 2022 Dec 14;23(24):15889. doi: 10.3390/ijms232415889 (PMC9785488; doi:10.3390/ijms232415889)
Supplement: Supplementary file 1 [file ijms-23-15889-s001.zip › ijms-2046697-supplementary.pdf]

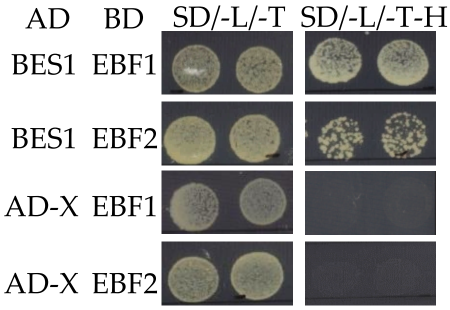

Figure S1. EBF1/2 interacted with BES1.

The yeast two hybrid assay showed BES1 interacted with EBF1 and EBF2 in yeast.

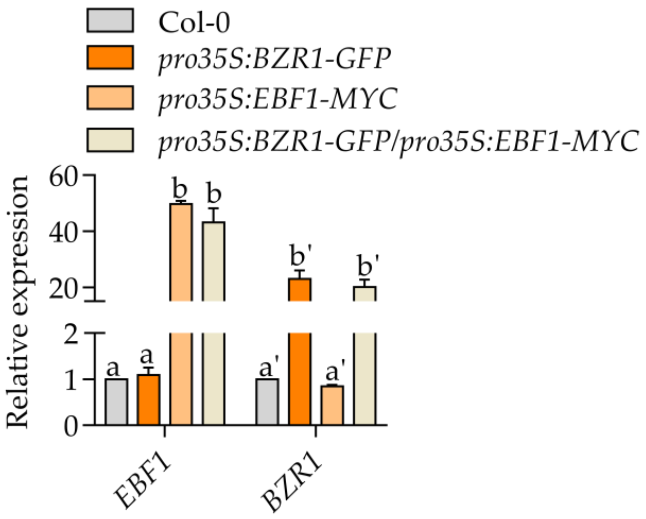

Figure S2. The expression level of *BZR1* and *EBF1* in the hybrid line.

Quantitative RT-PCR showed the *EBF1* and *BZR1* expression level in indicated plants. Total RNAs were extracted from 7-day-old seedlings grown on ½ MS medium. *PP2A* gene was analysed as an internal control. Error bars are SD of three biologic replicates. Different letters above bars indicate statistically significant differences between samples (One-way ANOVA followed by post-hoc Tukey test, $p < 0.05$).

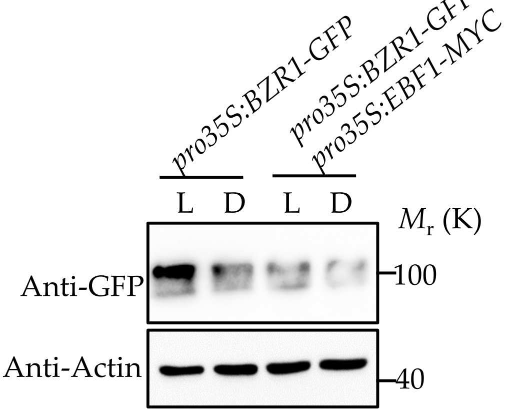

Figure S3. EBF1 promoted the degradation of BZR1 in darkness.

The protein level of BZR1 in transgenic Arabidopsis plants expressing *pro35S:BZR1-GFP* only or co-expressing *pro35S:EBF1-MYC* and *pro35S:BZR1-GFP*. Seedlings were grown on ½ MS medium for 7 days in in white light then treated with or without darkness for 24h. BZR1 was detected with anti-GFP antibody. Actin was used as a control.

Table S1. Oligo primers Used in this Study

Gene cloning

Gene	Forward	Reverse
EBF1	ATGTCTCAGATCTTTAGTTTTGCC	GGAGAGGATGTCACATTTGTAAAG

CHIP-PCR

Gene	Forward	Reverse
PP2A ChIP	CGGCTTTCATGATTCCCTCT	GCCTTAAGCTCCGTTTCCTACTT
EBF1 CHIP	GAACCCCAATTCTCAGCTC	CGAGGAGATAGAGAGCGAAG

RT-PCR

Gene	Forward	Reverse
PP2A RT	TTCTCGCTCCAGTAATGGGATCCGA	GTTCTCCACAACCGCTTGGTCGACT
SAUR15 RT	TTGAGGAGTTTCTTGGGTGCTAAG	GCCATGAATCCTCTTGGTGTCG
EXP8 RT	TCCTCCTCTTCAGCATTTCGACCT	CTTGCCACGACTGTGTTTTTGAGC
EBF1 RT	AGCAAACTGCCAGATTCTCAGTG	AGGAGGCCAATGCTTGAATCCC
BZR1 RT	GCAGATGTCTCCAAATACTGCTGCCT	GACATGCCATTTGGGTTTGCCTAG

Genotyping

Gene	Forward	Reverse
ebf1	AGCAATTGCTGTTGGAACTGCTGGT	TTCTCATGAACTGTGGCTCAA
LBI	GGCAATCAGCTGTTGCCCGTCTCACTGGTG	
pif1	CTCTTTTGGATCTTTCTGGGG	GACTTGCGCACGATAGCTAAC
pif3	AGTCTGTTGCTTCTGCTACGC	AAGAACCGGCAAAGATACCAC
pif4	AATTCATCATCGGGGATTAGG	TCGTCGTTTAATAAACACGGC
pif5	CGATTTGTTACCCATGGTTTG	CCTTGCTCGATTTTTGTTACG
LBb1.3	ATTTTGCCGATTTCGGAAC	
ein3-1	GAGCAAGCTAGGAGGAAGAAAATGTCTAG	TTTAGGCAAACCAAGTTGATGCCAC
eil1	GATCGTAATGGTCCAGCT	ATAAAGGATCCGAGCCACAACCTCTTC
